# NSAID Use and Effects on Pediatric Bone Healing: A Review of Current Literature

**DOI:** 10.3390/children8090821

**Published:** 2021-09-18

**Authors:** Stephanie Choo, Julia A. V. Nuelle

**Affiliations:** Department of Orthopaedic Surgery, Missouri Orthopaedic Institute, University of Missouri, Columbia, MO 65212, USA; slcdn4@health.missouri.edu

**Keywords:** NSAIDs, ibuprofen, fracture healing, pediatrics, evidence-based recommendations

## Abstract

This systematic review evaluates and synthesizes the available peer-reviewed evidence regarding the impact of non-steroidal anti-inflammatory drugs (NSAIDs) on fracture healing in skeletally immature patients. Evidence supports the use of NSAIDs in this patient population for adequate pain control without increasing the risk of nonunion, particularly in long bone fractures and pseudoarthrosis after spine fusion. However, further clinical studies are needed to fill remaining gaps in knowledge, specifically with respect to the spectrum of available NSAIDs, dosage, and duration of use, in order to make broad evidence-based recommendations regarding the optimal use of NSAIDs during bone healing in skeletally immature patients.

## 1. Introduction

Nonsteroidal anti-inflammatory drugs (NSAIDs) have consistently been some of the most widely used medications for decades, based on their anti-inflammatory and analgesic mechanisms of action, in conjunction with their safety profiles and non-addictive characteristics [1]. The opioid epidemic has generated a strong impetus for the use of multimodal pain regimens, aimed at effectively managing pain with less reliance on opioids and the associated risks of misuse, abuse, addiction, morbidity, and mortality [2,3,4]. Decreased reliance on opioids also mitigates the known side effects of this class of medication, including nausea, vomiting, dizziness, and constipation. As such, NSAIDs provide an attractive choice as a mainstay of multimodal pain management protocols. There is hesitancy, however, to prescribe NSAIDs within the period following a fracture, arising from concern around delayed healing or non-union, given the potential blockade of the required inflammatory phase of bone healing.

Fracture nonunions are painful, can negatively impact the patient’s quality of life, and are costly to the healthcare system [5]. In general, the incidence of fracture nonunion has been reported to range from 5% to 10% in the skeletally mature patient population, with even higher rates associated with at-risk patients and fractures of the scaphoid (15.5%), tibia and fibula (14%), and femur (13.9%) [6,7]. Pseudoarthrosis in the pediatric population is substantially less, at a rate of <1% [8]. Several animal models and retrospective cohort studies have demonstrated the potential for NSAIDs to increase the risk of non-union in the skeletally mature population [6,9,10]. However, recent prospective studies have shown that NSAIDs do not increase the risk of nonunion, while improving pain control with a potential opioid-sparing effect [11,12,13]. In addition to these contrasting results in skeletally mature population studies, evidence-based recommendations regarding the use of NSAIDs for pain management after fractures in the pediatric, skeletally immature population have similarly not been conclusive to date.

Fracture risk in pediatric patients is high, however, the incidence for nonunion is relatively low [8]. Still, while NSAIDs are highly desirable for effective pain management with a reduction in opioid use in this patient population, there has been an apparent hesitancy in prescribing NSAIDs to pediatric patients with fractures due to physicians’ concerns for non-unions based on some of the published data for adults. The concerns, based on anecdotal extrapolation to the pediatric population, does not have a sound physiologic basis in light of distinct differences in bone healing between patients that are skeletally immature and skeletally mature [14], and may unnecessarily inhibit best practice. Therefore, the purpose of this study was to (1) systematically review available evidence regarding skeletally immature fracture healing patterns based on exposure to NSAIDs to synthesize recommendations on NSAID use for pain control post-injury, and (2) determine critical gaps in knowledge towards establishing evidence-based care for analgesia after fractures in the pediatric population.

## 2. Materials and Methods

Using PRISMA guidelines, two electronic databases—PUBMED and Cochrane—were systematically searched using combinations of the keywords “NSAIDS”, “ibuprofen”, “nonsteroidal”, “anti-inflammatory drugs”, “naproxen”, “ketorolac”, “bone healing”, “fracture healing”, “bone fractures”, “bone union”, “non union”, “children”, “pediatric”, “child”, and “skeletally immature” as terms, title words, and abstract words. Reference lists from identified articles were also reviewed for inclusion. Studies in the English language and published between the years 2000–2021 were considered for inclusion. Studies were included when they assessed NSAID effects on bone healing in individuals who are skeletally immature and/or evaluated pediatric outcomes after NSAIDs use in association with fracture management. The exclusion criteria, included fracture or bone healing following orthopaedic surgical intervention not included as a study outcome, as well as studies of patients who are skeletally mature. The title and abstract of each qualifying article were screened and full-text manuscripts were retrieved in cases of uncertainty for inclusion. The following data from included studies were then extracted: Date of publication, number of patients, number of males and females, mean age, the incidence of nonunion or pseudoarthrosis, length of follow-up, type and amount of medication used, and the study type/level of evidence. An independent reviewer determined the final eligibility of studies included for systematic review. The synthesis of included articles followed the PICO (Population, Intervention, Comparison, Outcome) reporting methodology. The Cochrane Risk of Bias Tool was also utilized to assess and report bias risk.

## 3. Results

**Figure 1:**
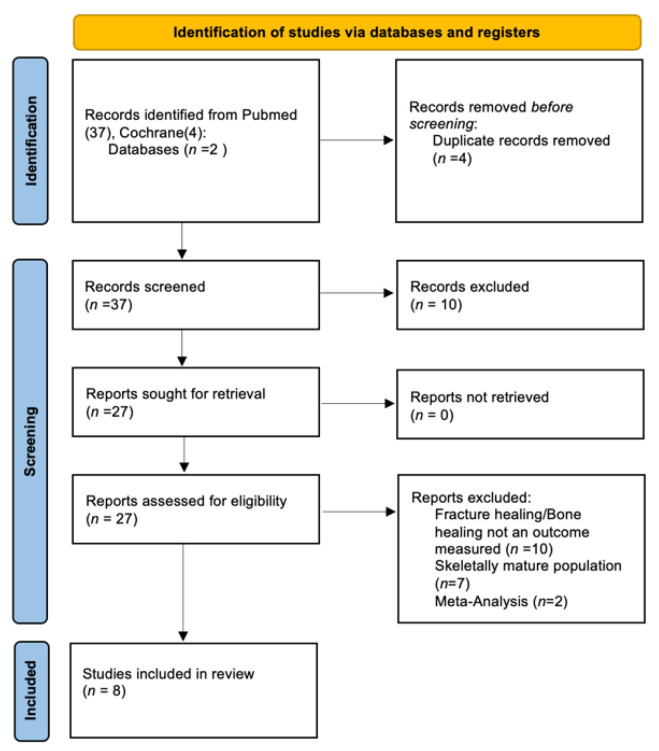
PRISMA 2020 flow diagram for new systematic reviews which included searches of databases and registers only.

The database search produced a total of 37 studies for review. A reference lists review produced an additional 4 relevant studies. Based on a full review of these 41 articles, 8 met inclusion criteria for systematic review (Figure 1).

From these 10 studies, 6 were retrospective cohort studies, 2 were prospective randomized control trials, and 2 were meta-analysis. The meta-analysis studies were excluded from this analysis as the 4 retrospective pediatric studies, cited in this literature, are also included in this current review. Demographic data for each study are included in Table 1. Qualitative synthesis, including risk of bias assessment for these 10 studies, were reported in Table 2.

### 3.1. Retrospective Cohort Studies

Vitale et al. [15] conducted a retrospective study to determine whether an association exists between the use of ketorolac and postoperative complications in children undergoing spinal fusion surgery for scoliosis. Ketorolac was shown to be an effective alternative to opioid medications for analgesia [21]. This study investigated potentially related complications, including bony fusion failure and the requirement for revision surgery due to pseudoarthrosis, as well as infected hardware, hardware failure, and bleeding risk represented by need for transfusion. Based on the review of 10 years of medical records, a total of 208 children who had undergone corrective spinal fusion surgery for scoliosis were included. Sixty patients (29%) received ketorolac postoperatively and the remaining 148 (71%) did not. The group of patients that were given ketorolac in the post-operative period was composed primarily of complicated patients (typically a greater degree of curvature) as determined by the operative surgeon. They reported an average dose of ketorolac to be 0.5 mg/kg intravenous (IV), every 6 h for 2–3 days, starting 24 to 48 h postoperatively. Patients were followed through clinical and radiographic assessments for an average of 62 months (ketorolac group) and 69 months (no ketorolac group). Analyses from this study produced no statistically significant differences between ketorolac versus no ketorolac for fusion rate, pseudarthrosis, or other potentially related complications. The only independent variable that was significantly associated with the need for revision surgery, due to pseudoarthrosis, hardware failure, or hardware infection was the degree of curvature of the spine prior to intervention.

The study by Sucato et al. [13] referenced a study by Glassman and colleagues, which demonstrated the significant inhibitory effects of ketorolac on spinal fusion when used as an adjunct for postoperative pain in adult patients [22], and served as the impetus for the former authors’ similar study in a pediatric population. This retrospective study compared patients who had postoperative ketorolac and those who did not, following posterior spinal fusion and instrumentation for adolescent idiopathic scoliosis (AIS). Overall, 161 patients did not receive ketorolac postoperatively and 158 did. Pseudoarthrosis was defined by surgical exploration and confirmation in the patients who presented with ongoing symptoms after initial fixation. This study noted the average number of ketorolac doses was 6.7 for a duration of 48 h after surgery. Patients in the ketorolac group augmented their medical pain management with an additional 5.8 doses of ibuprofen after surgery on average in comparison to the non-ketorolac group’s average of 0.7 doses. Mean follow-up for both groups was 39 months after fixation and the overall incidence of pseudoarthrosis during this time was 2.5% (8 of 319 patients) with no statistically significant difference between the ketorolac and non-ketorolac groups (1.9% vs 3.1%). The data from this study led the investigators to conclude that pseudoarthrosis risks are similar for AIS patients, regardless of ketorolac use, a finding consistent with those of Vitale et al. [15].

Kay et al. [16] performed a retrospective review of 221 children who sustained fractures (supracondylar humerus (42%), lateral condyle (12%), forearm (10%), femur (18%), tibia (7%), or ankle (7%)) requiring operative fixation. Of the 221 patients, 169 were treated with ketorolac postoperatively and 52 were not. The decision to use ketorolac for treatment was based on the preference of the anesthesiologist or the pain management team. The ketorolac group received weight-based dosing every 6 h while in the hospital, and the non-ketorolac group was treated with ibuprofen. These patients, on average, stayed 2.5 days in the hospital, discharged with ibuprofen, and followed for an average of 6.2 ± 7.5 months after fixation. No complications related to ketorolac or ibuprofen administration were reported, and there were no cases of delayed union or malunion in this pediatric fracture cohort.

In their 2011 study, Kay et al. [17] performed a retrospective review of 327 children undergoing lower extremity osteotomy surgery, of which 299 patients received ketorolac perioperatively and 28 patients did not. Delayed union occurred in 5 of 682 osteotomies (0.7%), which included 0.6% in the ketorolac group and 1.8% in the non-ketorolac group (*p* = 0.893). No nonunions were documented. The data in this study led the investigators to conclude that perioperative ketorolac is acceptable for pain management in patients who are skeletally immature and undergoing lower extremity osteotomies without significant risks regarding bone healing.

Zura et al. [8] performed a retrospective cohort study of 237,033 fractures in pediatric patients to identify risk factors for nonunion. Data were acquired from Truven Health Analytics health claims in a single calendar year (1 January 2011—31 December, 2012). The study population maintained a low nonunion rate until age 11 where a strong inflection point occurred such that a significant increase in nonunion rate was noted for each fracture location analyzed (*p* < 0.001), with tibia, fibula, femoral neck, and scaphoid having >5% nonunion risk after age 11. Interestingly, this study reported that pediatric fracture patients that used NSAIDs with opioids (*p* < 0.01; OR = 2.52) or opioids alone (*p* < 0.01; OR = 2.47) had a nearly 2.5 times increased risk of non-union, while patients (age 0 to 18 years) who consumed NSAIDs alone did not show a statistically significant increase in risk. This study did not specify which NSAIDs were used, or the dosage and duration of use. This data led the authors to conclude that NSAID use is appropriate for pain management during bone healing in pediatric patients, but should not be used in conjunction with opioids, especially after age 11.

DePeter et al. [18] performed a retrospective study in children aged 6 months to 17 years, assessing patients with common fractures that have been reported to have a higher incidence of healing complications, including in the tibia, femur, humerus, scaphoid, and fifth metatarsal fractures. A total of 808 patients were included in this study, of which a total of 27 (3%) showed bone healing complications, 1% from nonunion, 0.4% delayed union, and 2% loss of reduction. Of the 338 patients (42%) exposed to ibuprofen, 10 patients (3%) went on to non-union, which was not significantly (*p* = 0.61) different from patients not consuming ibuprofen. These data led the authors to conclude that there was no significant association between ibuprofen use and the development of bone healing complications in this pediatric high-risk fracture population.

### 3.2. Prospective Randomized Studies

Nuelle et al. [20] performed a randomized, parallel, single-blinded clinical trial to assess the impact of NSAIDs on acute-phase bone healing in patients who are skeletally immature with a variety of long bone fractures. This study assessed 97 fractures in pediatric patients, randomized to control (*n* = 47) or NSAID (*n* = 50) cohorts. The control group was provided weight-based acetaminophen for pain control and oxycodone for breakthrough pain. If a patient was in the emergency department, weight-based fentanyl was available for administration. Patients in the NSAID group were prescribed weight-based ibuprofen for pain control, as well as oxycodone for breakthrough pain. NSAID patients in the emergency department were permitted to have ketorolac for pain control if needed. Clinical visits involving radiographs of the fractured extremity occurred at 1–2 weeks, 6 weeks, 10–12 weeks, and 6 months after injury. Fracture healing was determined by clinical examination, absence of tenderness to palpation at fracture site, and radiographs demonstrating callus formation bridging in 3 cortices. As a secondary outcome measure, pain control was assessed by the patient’s caregiver recording the patient’s visual analog scale pain score, as well as the amount and type of pain medication used, for a total of 3 weeks after the injury. This study demonstrated that, by 6 weeks following initial fracture treatment, 82% of the control group had united fractures and 92% of the NSAID group had healed fractures, with no significant difference (*p* = 0.22) between both groups. By the 10–12 week appointment, 98% of control and 100% of NSAID patients had healed fractures, and the 6-month follow-up demonstrated 100% healed fractures for both cohorts. Of note, pain scores were similar for both groups with acetaminophen used for 3.9 days on average versus ibuprofen use for 4.3 days on average. Oxycodone was used for breakthrough pain for a mean of 2.4 days in the control cohort and 1.9 days in the NSAID cohort. These data led the authors to conclude that ibuprofen can be used to effectively manage pain in pediatric patients during the acute post-treatment period following long bone fractures without impairing fracture healing.

Drendel et al. [19] performed a randomized, double-blind, clinical trial assessing ibuprofen versus acetaminophen with codeine in pediatric patients treated for upper extremity fractures. In contrast to Nuelle et al. [20], the primary outcome measure for this study was pain control, with fracture healing as a secondary outcome measure. Pain was assessed using the visual Bieri Faces Pain Scale and caregivers were also asked to keep a diary documenting the patient’s tolerability of the medication. Non-unions were assessed by reviewing medical records for 1 year following treatment, with a follow-up phone call to the patient’s caregiver for verification when an adverse event related to fracture healing was noted. The study enrolled 336 children, and 169 patients were randomized to the ibuprofen group and 167 to the acetaminophen with codeine group. Of the 244 patients that met the criteria and were analyzed, the proportion of “treatment failures” and the need for rescue IV pain medication was lower in the ibuprofen group than the acetaminophen with codeine group (20.3% vs 31.0%, respectively). However, this difference was not statistically significant. Pain scores were similar between the two groups. However, medication-related adverse effects reported during the first 3 days of treatment were significantly higher for the acetaminophen with codeine group at 50.9% versus the ibuprofen group at 29.5% (% difference 21.4, confidence interval (CI) 9.1 to 33.7). Chart review and telephone calls determined that four (1.6%) of the children sustained re-fracture within 1 year of the original fracture, and of those, three (75%) received acetaminophen with codeine. There were no reports of nonunion in either group. These data led the authors to conclude that ibuprofen was as effective as an acetaminophen with codeine in providing analgesia for children with upper extremity fractures. The drug was associated with less medication-related adverse effects and was not associated with re-fracture or nonunion in this patient population.

## 4. Discussion

This systematic review of available evidence regarding NSAID exposure, and bone healing in patients who are skeletally immature, produced six retrospective studies and two prospective clinical trials for inclusion. The qualitative synthesis of these studies suggested that the best current evidence supports the use of two NSAIDs, ibuprofen and ketorolac, as safe and effective medications for pain management. These medications did not show an increased risk of pseudoarthrosis after spinal fusions or nonunion after fractures of the upper and lower extremity, scaphoid, or metatarsals in the pediatric population.

Concerns regarding the use of NSAIDs in the acute phase of bone healing have been based on animal studies and clinical assessments of patients that are skeletally mature. To date, there have been no studies demonstrating that NSAID use in the acute phases of bone healing result in higher rates of nonunion.

Bone healing involves a complex cascade of events with the end-goal being the formation of bridging vascularized woven bone capable of functional remodeling over time [23]. Studies have indicated that prostaglandins, which are important in the regulation of osteoclastic bone resorption and osteoblastic bone formation, required for bone healing, are released at the time of fracture via cyclooxygenase enzyme activity (COX-2) [8]. As COX inhibitors, NSAIDs can arrest the cell cycle and increase cytotoxicity and apoptosis of osteoblasts, leading to ineffective mineralization of newly formed extracellular bone matrix [24]. In studies of skeletally mature animals [25,26,27,28], non-selective NSAIDs were shown to delay long bone healing without any detrimental effects on long-term outcomes. Whereas selective NSAIDs were associated with detrimental long-term effects on material and structural properties of healing bone. Research that focused on dose and time effects suggested avoiding NSAID treatment during the initial weeks of fracture healing, in order to avoid these negative effects on fracture callus [7]. However, the physiology of the skeletally immature system is distinct from the skeletally mature. Some contributors to this unique physiology, which may make skeletally immature patients less prone to pseudoarthrosis, include thicker periosteoum, greater subperiosteal hematoma, active physes, and enhanced metabolic activity. Capello et al. explored the effects of NSAID administration during the acute phase of fracture healing in skeletally immature animals, and found no significant difference in strength, stiffness, or histologic characteristics of fracture callus. These finds support the view that the bone healing physiology of the skeletally immature is not perturbed by NSAID administration at physiologic doses [29].

A large inceptive cohort study by Zura et al. demonstrated the combination of NSAIDs and opioids (multivariate odds ratio (OR), 1.84; 95% CI, 1.73–1.95) is a risk factor for nonunion. However, this study found no significant association of NSAIDs alone with nonunion (multivariate OR, 0.98, 95% CI, 0.89–1.07, *p* = 06), but found opioids alone to be a moderately strong positive risk factor for nonunion (multivariate OR, 1.43, 95% CI, 1.34–1.52, *p* ≤ 0.001) [6]. Zura et al. expanded on this original study to isolate pediatric patient risk factors for nonunion and found similar results, further supporting the finding that NSAIDs are not significant contributors to fracture nonunion, but NSAIDs combined with opioids, lead to significant risks [8].

This analysis is limited by the number of studies available, as well as heterogeneity of the bone healing types included in the reviewed studies, including fractures treated operatively compared with those treated by closed means, osteotomies, as well as spinal fusions. As with all reviews, this review is an analysis of the best available published data. Certainly, future studies on the potential multicenter participation and non-inferiority designs, examining each of these bone healing types, are warranted. More robust data are needed to assess the impact of NSAID use on bone healing in the skeletally immature population.

## 5. Conclusions

Clinical data investigating the effects of NSAIDS on bone healing in the pediatric population is sparse. The retrospective studies of pediatric patients who used NSAID following posterior spinal fusion did not demonstrate inhibition of bone healing [13,15]. Two studies by Kay et al. found no cases of nonunion in pediatric patients who received ketorolac around the time of operative fixation of common pediatric fractures or lower extremity osteotomy [16,17]. DePeter et al. similarly found no healing complications arose for the pediatric population exposed to ibuprofen during fracture healing. The two prospective studies by Nuelle et al. [20] and Drendel et al. [19] support the use of ibuprofen for long bone fractures in the acute healing period for adequate pain control without compromising bone healing. In conclusion, based on the current available evidence, NSAID use in the acute phase of bone healing in skeletally immature patients is not associated with a higher rate of pseudoarthrosis.

## Figures and Tables

**Table 1:** Demographics.

Study Year	Author	Number of Patients	Male/Female	Mean Age (Not Exposed/Exposed)	Mean Follow Up (Not Exposed/Exposed)
2018	Zura et al. [8]	237,033 fractures	146,234 fractures/90,790 fractures	0–18 years	12 months
2008	Sucato et al. [13]	319 total (158 exposed)	50/269	14.2 years	39 months
2003	Vitale et al. [15]	208 total (60 exposed)	59/149	13.4 years	67 months
2010	Kay et al. [16]	221 total (169 exposed)	142/79	6.7 years (5.5 years/7.1 years)	6.2 months (6.7 months/6.9 months)
2011	Kay et al. [17]	327 total (299 exposed)	181/136	(8.4 years/9.2 years)	(38 months/44 months)
2016	DePeter et al. [18]	808 total (338 exposed)	508/300	7 years (7 years/7 years)	--
2009	Drendel et al. [19]	336 total (169 exposed)	126/210	(8.2 years/7.4 years)	4 years
2020	Nuelle et al. [20]	95 total (49 exposed)	58/37	7.6 years	6 months

**Table 2:** Synthesis of Examined Studies Evaluating Effect of NSAIDs on Bone Healing in Skeletally Immature Patients.

Study	Fracture Locations	NSAID Used	Clinical Relevance/Findings	LOE/Study Type	Risk for Bias
Drendel et al. [19]	Upper extremity fractures	Ibuprofen	Primary outcome: Ibuprofen was at least as effective as acetaminophen with codeine as outpatient analgesia for children with arm fractures Secondary outcome: No nonunions reported with use of ibuprofen or acetaminophen with codeine	I—RCT	Low risk
Nuelle et al. [20]	Long bone fractures	Ibuprofen and Ketorolac	Ibuprofen does not impair clinical or radiographic long bone fracture healing in skeletally immature patients	I—RCT	Low risk
Zura et al. [8]	Metacarpal, radius, ankle, patella, radius and ulna, fibula, pelvis, clavicle, humerus, femur, tibia, ulna, metatarsal, tarsal, tibia and fibula, scaphoid	Did not specify	NSAIDs alone did not increase risk of pediatric nonunion Risk factors for pediatric nonunion are similar to adult nonunion risk factors [increasing age, male gender, high body-mass index, severe fracture (e.g., open fracture, multiple fractures), and tobacco smoking] Opioids should be used cautiously in pediatric patients, as they are associated with a significant and substantial elevation of nonunion risk	II—Prognostic Retrospective Study	Moderate risk
DePeter et al. [18]	Tibia, femur, humerus, scaphoid, or fifth metatarsus	Ibuprofen	There was no statistically significant association between ibuprofen exposure and the development of a bone healing complications in the tibia, femur, humerus, scaphoid, or fifth metatarsal	III—Retrospective Comparative Study	Moderate risk
Sucato et al. [13]	Vertebrae	Ketorolac	Ketorolac does not increase the incidence of developing a pseudoarthrosis when used as an adjunct for postoperative analgesia following a PSFI for AIS using segmental spinal instrumentation and iliac crest bone graft.	III—Retrospective Comparative Study	Moderate risk
Kay et al. [16]	Supracondylar, forearm, lateral condyle, femur, tibia, or ankle fractures	Ketorolac and ibuprofen	Perioperative ketorolac use does not increase the risk of complications after operative fracture care of supracondylar, forearm, lateral condyle, femur, tibia, or ankle fractures in children	III—Retrospective Comparative Study	Moderate risk
Kay et al. [17]	Lower extremity osteotomies	Ketorolac	Perioperative ketorolac is safe for children having lower extremity osteotomies as there is no significant difference in the rate of either osseous or soft tissue complications between ketorolac provided and no ketorolac patients	III—Retrospective Comparative Study	Moderate risk
Vitale et al. [15]	Vertebrae	Ketorolac	Degree of spine curvature is significant in predicting reoperation; treatment with ketorolac is not a significant independent predictor for nonunion/reoperation	III—Retrospective Comparative Study	Moderate risk

LOE = Level of Evidence; RCT = Randomized Controlled Trial.
